# Using Information Technology and Social Networking for Recruitment of Research Participants: Experience From an Exploratory Study of Pediatric Klinefelter Syndrome

**DOI:** 10.2196/jmir.2286

**Published:** 2013-03-19

**Authors:** Sharron Close, Arlene Smaldone, Ilene Fennoy, Nancy Reame, Margaret Grey

**Affiliations:** ^1^Yale UniversitySchool of NursingNew Haven, CTUnited States; ^2^Columbia UniversitySchool of NursingNew York, NYUnited States; ^3^Columbia University Medical CenterDivision of Pediatric EndocrinologyNew York, NYUnited States

**Keywords:** patient recruitment, research subject recruitment, health information technology, social networking, Klinefelter syndrome

## Abstract

**Background:**

Recruiting pediatric samples for research may be challenging due to parental mistrust of the research process, privacy concerns, and family time constraints. Recruitment of children with chronic and genetic conditions may further complicate the enrollment process.

**Objective:**

In this paper, we describe the methodological challenges of recruiting children for research and provide an exemplar of how the use of information technology (IT) strategies with social networking may improve access to difficult-to-reach pediatric research participants.

**Methods:**

We conducted a cross-sectional descriptive study of boys between the ages of 8 and 18 years with Klinefelter syndrome. This study presented unique challenges for recruitment of pediatric participants. These challenges are illustrated by the report of recruitment activities developed for the study. We reviewed the literature to explore the issues of recruiting children for research using conventional and IT approaches. Success rates of conventional recruitment approaches, such as brochures, flyers in medical offices, and physician referrals, are compared with IT-based outreach. The IT approaches included teleconferencing via a Klinefelter syndrome support group, services of a Web-based commercial recruitment-matching company, and the development of a university-affiliated research recruitment website with the use of paid advertising on a social networking website (Facebook).

**Results:**

Over a 3-month period, dissemination of over 150 recruitment brochures and flyers placed in a large urban hospital and hospital-affiliated clinical offices, with 850 letters to physicians and patients were not successful. Within the same period, face-to-face recruitment in the clinical setting yielded 4 (9%) participants. Using Web-based and social networking approaches, 39 (91%) agreed to participate in the study. With these approaches, 5 (12%) were recruited from the national Klinefelter syndrome advocacy group, 8 (19%) from local and teleconference support groups, 10 (23%) from a Web-based research recruitment program, and 16 (37%) from the university-affiliated recruitment website. For the initial 6 months, the university website was viewed approximately 2 to 3 times per day on average. An advertisement placed on a social networking site for 1 week increased website viewing to approximately 63 visits per day. Out of 112 families approached using all of these methods, 43 (38%) agreed to participate. Families who declined cited either travel distance to the study site (15, 22%) or unwillingness to disclose the Klinefelter syndrome diagnosis to their sons (54, 78%) as the reasons for nonparticipation.

**Conclusions:**

Use of Web-based technologies enhances the recruitment of difficult-to-reach populations. Of the many approaches employed in this study, the university-affiliated recruitment website supported by a Facebook advertisement appeared to be the most successful. Research grant budgets should include expenses for website registration and maintenance fees as well as online advertisements on social networking websites. Tracking of recruitment referral sources may be helpful in planning future recruitment campaigns.

## Introduction

Recruiting children for research can present many challenges due to parental mistrust of the research process, privacy concerns, and family time constraints [1,2]. Children with chronic and genetic conditions may further complicate the recruitment process [3]. For studies conducted in the United States, additional challenges exist including regulations and guidelines that direct how researchers contact and enroll participants for studies.

Prior to 1996, medical treatment of children was based on clinical trials and the testing of products and medications that were conducted in adults [4]. Although many treatments were effective for adults, some were shown to be ineffective or harmful to children [4]. In 1996, members of a joint workshop of the American Academy of Pediatrics and the National Institute of Child Health and Development issued a consensus statement calling for children to receive adequately tested treatments recommending efforts to include children in research [4]. In1998, the National Institutes of Health (NIH) established a policy with guidelines requiring that children must be included in all human subjects research that is conducted or supported by the NIH unless there was a scientific or ethical reason for not doing so [5]. The goal of this policy was to increase child participation in research for the purpose of generating data specific to the treatment of children [4,5]. However, inadequate representation of children in selected areas of low-prevalence diseases, orphan conditions (such as Klinefelter syndrome), and genetic conditions persist [6]. More innovative approaches to the enrollment of pediatric volunteers in clinical studies are needed.

The general public increasingly uses information technology (IT) as a source of health information. Approximately 80% of the American public search for health information using Internet sources [7]. Researchers are now turning to the Internet as a tool to recruit target study populations [8-14] for Internet-based interventions in conditions such as hypertension [15], diabetes [16-18], smoking cessation [8,12,19], human immunodeficiency virus risk management [13], and depression [14].

The Internet offers many opportunities for informing potential research participants about a study. These include email [8,20,21], discussion boards, blogs [8], search engines [20,21], study websites [20-22], and Web-based platforms for matching researchers with participants. Each form of Web-based communication provides opportunities and challenges for subject recruitment.

On the one hand, exposure of information to vast numbers of Internet users creates an enormous opportunity for visibility and communication with potential research participants. At the same time, the recruitment process can be sabotaged by problems on the Internet, such as emails sent to spam folders [8-10], discussion board and blog administrators blocking content associated with the researcher [9], and poor choice or lack of adequate keywords on study websites that diminish search engine exposure [23]. A key limitation of the use of Internet-based recruitment activities include the inability to reach socioeconomically or educationally disadvantaged groups as well as culturally diverse populations who may lack access to the Internet or familiarity with its use [10]. In the same vein, potential recruits to a study may not be receptive to unsolicited emails, or may not trust the legitimacy of the sender [8]. Clinical investigators encounter special challenges when attempting to recruit children as research volunteers, especially those who have low-prevalence diseases or genetic conditions [6,24].

The purpose of this paper is to describe methodological challenges associated with the recruitment of children as volunteers in research and to discuss how IT may improve access and enrollment of children in research. A case study of our experience in recruiting boys with Klinefelter syndrome for an exploratory cross-sectional study is used to illustrate specific challenges encountered with a difficult-to-recruit pediatric research population and how IT was used to support the enrollment of participants in the study.

### Research Recruitment Challenges

Successful enrollment of clinical research participants is both a science and an art. A number of factors, including patients, health care professionals and researchers, structural and organizational entities, and history, interact to compromise or undermine successful enrollment of patient volunteers into a clinical study [1]. Patients may have limited access to research information and might not fully understand the role of clinical research in the advancement of knowledge for drug and behavioral therapy development [1,21]. They may also worry about or mistrust researchers and their institutions due to lack of understanding about the research process or associated risks and benefits [1,21,25]. Patient characteristics, such as culture, language, and religion [1], may further reduce the chance of successful enrollment. Health care providers may play an important role in gaining access to potential participants, but also may represent barriers to such access [1,2,21].

Health care providers in nonacademic settings may have a limited understanding or interest in clinical trials or may have misgivings about academic institutions [1,26]. Community health care providers may also have concerns about losing control over their patient’s care, or losing the patient to another provider [1,26]. Full-time clinicians are frequently pressed for time in caring for patients and may be concerned about the additional administrative workload and lack of administrative support for research activities [1,2,26]. This concern may lead to financial disincentives for clinical providers to become involved in informing their patients about research enrollment opportunities [26]. Researchers themselves sometimes fail to recognize how they may contribute to recruitment and enrollment problems in their own studies. Lack of training and proficiency in communication for the conduct of research with low-literacy populations may lead to misunderstandings between the researcher and potential participant and, in turn, lower response rates of participation [1].

Other barriers facing researchers include lack of attention to the mistrust of the population to be recruited, failure to demonstrate cultural sensitivity, and lack of training in understanding health care disparities in underserved populations [1]. These barriers are often unrecognized by researchers and get in the way when attempting to gather the desired sample. Structural and organizational factors may also be associated with the desire or ability of people to volunteer for research [1]. Researchers need to consider logistic arrangements to facilitate patient participation, such as creating convenient times and locations for study participation.

Communities may also be sensitive about allowing researchers entry into their environment, especially when they perceive that their participation in the scientific efforts does not result in any return or reward at the community level [1]. This concern makes it very difficult for researchers to re-enter the same community or for the community to be approached by other researchers.

The history of disreputably negative research practices persists in the minds of the public and these perceptions may influence the attitudes of potential research volunteers. The awareness of inhumane treatment by Nazi researchers during World War II and the infamous Tuskegee Syphilis Project conducted by the US Public Health Service from 1932 to 1972 [27] may promote overall fear and mistrust about the research process in the minds of many potential research participants.

### Levels of Protection that Challenge Research Recruitment

In the United States, several guidelines offer protection to the public with regard to personal health care and participation in research. Public Law 104-191, also known as the Health Insurance Portability and Accountability Act (HIPAA), was enacted to protect the privacy and personal health information of the public [28]. The HIPAA requirements also guide researchers on how to protect the privacy of research participants. Although all health care providers and researchers are required to obey these laws, many members of the public may be apprehensive of the attendant side effects of disseminating private medical information by researchers. Levels of protection, designed to benefit the public, also may impede progress in the timing and accomplishment of recruitment.

Although pediatric researchers are charged with the responsibility of recruiting children for research, several challenges exist in such efforts. Because parents are legally responsible for their children, it is the parent who must be approached for permission for their child to participate. Parents’ willingness to have their child participate in a study may be influenced by their perception of benefits, risks, and barriers to participation [2]. The child must also assent to the activities of the research project. The child’s willingness to participate in the project may depend upon his/her developmental status and any vulnerability related to illness, chronic condition, or communication disabilities. Children may view research participation as a positive experience, including a wish to help others, reward incentives, and the desire to have a fun experience [2]. These positive motivations may be offset by anticipated unpleasantries, such as blood tests, disagreeable medication regimens, or interruptions in their daily lives [29]. The child-recruit is embedded within a family with complex daily schedules often including parental work, school schedules, and sport practices or other extracurricular activities. All members of the family, including the child’s siblings, influence the busy family schedule. Researchers must anticipate and accommodate time commitments of the family as well as considerations for transportation and commute time. Finally, the parents and the child-recruit must be prepared to agree about certain participation risks and unpleasantries such as completing multiple forms and surveys, or medical examinations, including blood collection.

## Methods

### Case Illustration: A Study of Boys With Klinefelter Syndrome

The exemplar case illustrates our recent experience with recruiting boys with Klinefelter syndrome for participation in a cross-sectional study. Traditional approaches to recruitment fell short of obtaining the desired sample and expanding the approach with IT resulted in a significant gain in enrollment.

Klinefelter syndrome is a genetic condition caused by the presence of an extra X chromosome (karyotype 47, XXY). This condition occurs in an estimated 1 in 450-500 male births [30,31]. Although it is not rare, it is extremely underdiagnosed. Approximately 64% of affected males are not aware of the diagnosis, and of the 36% who are aware, only 10% are diagnosed in childhood [32]. Klinefelter syndrome in adults is associated with androgen deficiency, gynoid distribution of body fat, gynecomastia, small testes, and azoospermia [33,34]. Individuals diagnosed with Klinefelter syndrome during adulthood report childhood developmental delay; speech, language and learning problems; and psychological issues including depression, shyness, aggression, and social interaction difficulties [35,36]. Klinefelter syndrome poses increased health risks throughout the life span, including increased risk for cardiovascular disease, diabetes, and osteoporosis [37]. Diagnosis of Klinefelter syndrome during childhood may represent an opportunity to address both physical and psychosocial health challenges.

Klinefelter syndrome is a misunderstood condition owing to a paucity of research in children, lack of clear clinical guidelines for treatment during life stages, and unfortunate conclusion errors made by early researchers that suggested men with Klinefelter syndrome were at increased risk for criminal behavior [38-40]. As a result, Klinefelter syndrome families may struggle with inadequate information, lack of support, perceived stigma, and uncertainties about their son’s health [41]. Current research focused on boys with Klinefelter syndrome report fairly small sample sizes, ranging from groups of less than 20 [42-44] to the largest reported cohort of 93 [45]. Misunderstandings about Klinefelter syndrome may contribute to reluctance on the part of many men and families of young sons with Klinefelter syndrome to discuss or disclose information about their diagnosis to others [24].

We conducted an exploratory descriptive study to better understand phenotype, biomarkers, and psychosocial health parameters of boys with Klinefelter syndrome between the ages of 8 and 18 years [46]. The study protocol included a physical examination, blood collection for reproductive and cardiovascular biomarkers, and psychosocial health measurements including quality of life, self-esteem, self-concept, and risk for depression. The Columbia University Institutional Review Board approved the protocol for this study. For this exploratory study, sample size was based on a moderate correlation of at least 0.40 between the clinical characteristics and psychosocial variables as observed in studies of health-related quality of life and polycystic ovary syndrome [47,48]. For a correlation of 0.40 with alpha=.05, a total of 46 subjects were required for a minimum power of 80%. No previous studies with a Klinefelter syndrome population studied the relationship between clinical characteristics and psychosocial health. Recruitment was planned with traditional approaches, including contacting patients in a local pediatric endocrine practice; sending letters to pediatricians, pediatric endocrinologists, geneticists, and genetic counselors; and the use of recruitment flyers and brochures placed strategically throughout the medical center. After sending 850 letters, placing 150 brochures and fliers, and approaching 23 families during clinical visits, only 4 boys were recruited in a 3-month period. It became readily apparent that the traditional approach would fail to achieve the minimum sample size of 46 according to our sample size calculation. Thus, a more innovative approach was devised using IT and social networking.

New recruitment strategies included the development of a study website, in-person information sessions, Web-links, teleconferences, and email access to members of a national and several regional Klinefelter syndrome support organizations, as well as registration with a computer platform clinical recruitment-matching service. Each strategy is briefly described subsequently.

#### Klinefelter Syndrome Study Recruitment Website

A study information and recruitment website [49] was created using the keywords *Klinefelter syndrome*, *KS*, *boys with KS*, and *KS phenotype* to increase the likelihood that people searching the Internet for information on Klinefelter syndrome might find the website when conducting searches. The website pages provided information regarding the study and its eligibility requirements, study procedures, and how to contact the researcher for further information or enrollment. The study website home page screenshot is shown in Multimedia Appendix 1.

#### Patient Advocacy Associations

We contacted a national Klinefelter syndrome advocacy association, Knowledge Support & Action (KS&A) [50], who agreed to place information about our study with the study website link on their website. A screenshot of KS&A home page is provided in Multimedia Appendix 2. Regional Klinefelter syndrome support groups with links to the national organization then invited us to give live presentations about our study at their meetings and also agreed to send emails about the presentation and the study to their members. One of the regional groups, the Klinefelter Syndrome Global Support Group (screenshot is shown in Multimedia Appendix 3), offered a monthly parent teleconference. Over a 3-month period, we were able to explain the purpose of the study and to respond to questions regarding our protocol.

#### Web-Based Clinical Recruitment-Matching Service

RecruitSource is a search engine and computer platform for matching clinical research participants with researchers [51]. A screenshot of the RecruitSource home page can be seen in Multimedia Appendix 4. Researchers can register details about their study and provide eligibility requirements for matching with potential participants.

Patients who might be interested in research participation register their health information via PrivateAccess [52] as shown in Multimedia Appendix 5. This website is a secure Internet registry that enables them to control who can and cannot see all or selected parts of their personal health information. This IT-based platform prescreens the potential participants who give advance privacy directives about their health information and are asked whether they wish to be contacted by a researcher. The incentive for people using this registry is that they can share their personal health information with properly authenticated doctors, researchers, or family members on a secure Internet platform. All contact information is coded and encrypted for privacy. The potential participant gives specific permission to be contacted by the researcher. Once the patient is registered, the researcher receives information about participants who have expressed an interest in being contacted for possible inclusion in the study. This service is provided at no cost to the researcher if the RecruitSource Web link is accessed via a patient advocacy association. In this case, the study was linked to the KS&A organization, a national advocacy association for Klinefelter syndrome [50].

#### Social Networking

Social networking is often defined by Web-based platforms, such as Facebook and others. Social networking, however, may also include face-to-face and teleconference transactions with groups, audiences, researcher-participant, and participant-participant networking. Participant-participant networking is the central component to the recruitment strategy known as *snowballing* [53]. We used all these networking processes in our Klinefelter syndrome study. The interlinking of IT-based and face-to-face networking provided an opportunity for multiple modes of information exposure about the study. Midway into recruitment, we decided to conduct a short trial of a Facebook advertisement (ad) as shown by the screenshot in Multimedia Appendix 6. Because we had not anticipated this strategy a priori, funding for advertising was limited. Nevertheless, we wished to observe how a 1-week social networking ad might impact exposure to the study website.

## Results

Of 112 families approached, 43 (38%) agreed to participate. The most frequent reasons for families declining participation was nondisclosure of the diagnosis to their sons (54/112, 78%) and geographic distance from the study site (15/112, 22%). Most parents who had not disclosed the diagnosis to their sons feared that their sons would learn of the diagnosis through participation.

Recruitment approaches for the participants in the Klinefelter syndrome study are summarized in Table 1. Recruitment using IT and social networking yielded a greater number of participants (39/43, 91%) compared to use of traditional approaches (4/43, 9%).

Of the 69 families who declined, over one-fifth (15/69, 22%) came from direct clinical contact; almost twice that number (29/69, 42%) declined during support group presentations, and one-sixth (10/69, 16%) declined during the national KS&A meeting. The most frequent reason for decline was parents not wanting their boys to learn of their diagnosis (n=54, 78%) and travel distance to study site (15/69, 22%).

**Figure 1:**
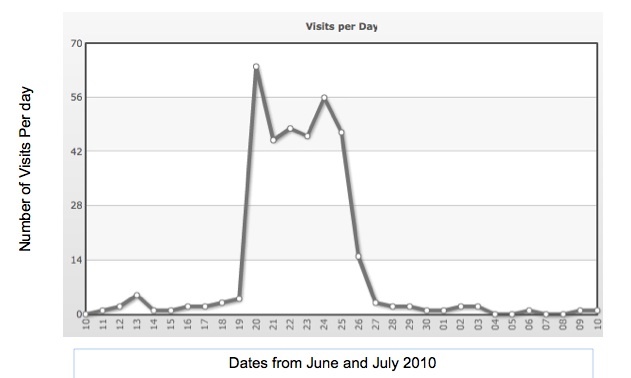
Response (visits per day to the Klinefelter syndrome study website) during Facebook advertisement period showing increase in activity during Facebook advertising.

In an effort to boost activity from general Web users, we placed an ad on Facebook. The ad ran for 1 week in June of 2010, targeting a general audience. Impressions are the raw number of times an ad is shown to different Facebook users. The Facebook ad was shown a total of 2,522,169 times. Social impressions reflect the number of times the ad was shown with social context who visited the study webpage. There were 2835 social impressions for this ad resulting in 509 clicks directly to the study website’s home page. At a total cost of $311 for the week’s ad, this represents the researcher’s cost of $0.61 per visit. Prior to placing the ad on Facebook, the study website received 2 to 3 visits per day. During the week of Facebook advertising, website visits climbed to an average of 63 visits per day. The Klinefelter study website activity increase in response to the Facebook ad can be seen in Figure 1.

**Figure 2:**
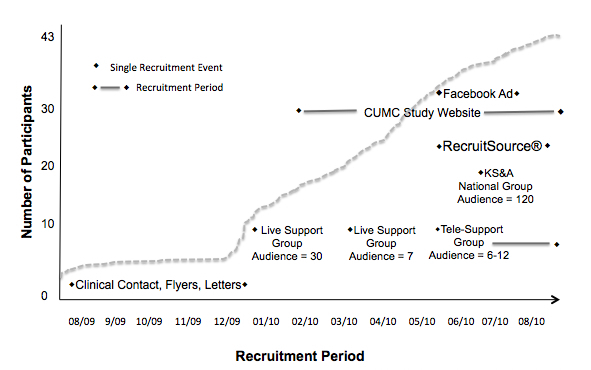
Recruitment to the Klinefelter syndrome study by source timeline.

Because multiple techniques were employed to attract this difficult-to-reach population, it is difficult to attribute any one recruitment approach to increasing the number of participants in this study. Figure 2 shows a timeline of the 1-year recruitment process.

**Table 1:** Number of participants using traditional and information technology with social networking recruitment approaches to the Klinefelter syndrome study (N=43).

Recruitment approach		n	(%)
**Traditional**		4	9
	Physician letters	0	0
	Patient letters	0	0
	Brochures	0	0
	Clinic referral	4	9
**Information technology and social networking**		39	91
	Advocacy group	5	12
	Support groups and teleconference	8	19
	RecruitSource	10	23
	Study website with Facebook ad	16	37

## Discussion

The Internet represents an increasingly valuable resource for researchers, especially for those who wish to understand the social and cultural context of the populations they are attempting to reach [54]. Since the advent of IT as a common mode of communication, researchers have learned many lessons about the pearls and pitfalls of using this recruitment approach. In difficult-to-reach and vulnerable populations, such as Klinefelter syndrome families, our experience has led us to understand better that more than 1 recruitment technique may be required to inform potential participants and to foster trust in them. We believe that the construction and launch of the study website served these 2 important purposes. We were, however, unable to attribute increased participation due to any 1 technique, including placement of the Facebook ad. Important lessons were learned during this challenging recruitment process, such as the need to track how participants make decisions about whether to participate. We were unable to track which potential research candidates came from the study website activity while the Facebook ad ran because we did not have access to the server log. Anecdotally, several families reported that they chose to participate only after being exposed to the study information from multiple sources. Some families reported that friends or other family members who saw the Facebook ad contacted them to let them know about the study. Once informed, these families conducted either a general Internet search, visited the website directly, or visited the KS&A website for more information. Most importantly, we discovered that we need to track sources of recruitment more carefully in the future by surveying participants about how they found out about the study and also by looking at server logs whenever possible. It would also be helpful to track website visits by Internet protocol (IP) addresses to examine how many potential candidates are first-time or repeat visitors. Because we were unable to attribute which of our recruitment responses came from the Facebook ad, we are unable to estimate the cost per participant. This information would have been very helpful in planning cost allocation for a future study.

Since the advent of IT and social networking in the scientific community, there has been a steady evolution of its use for recruitment and Internet-based interventions. Even within the past 5 to 7 years, much has been learned about the limitations of Internet-based approaches and how such problems might be mitigated.

Although early experience with the use of IT-based recruitment for clinical research, as reported by Koo and Skinner [8], was disappointing, others have offered solutions to optimize challenges that make this form of recruitment difficult. Murray et al [20] solved issues related to mass emailing and spam management by providing recipients with the option to unsubscribe in order to decline further contact by researchers. They were also able to demonstrate the benefits of advertising their study on the home page of a well-known and trusted charity. Our Klinefelter syndrome study recruitment was greatly enhanced by our exposure with the KS&A national advocacy association and with support groups. Ip et al [9] addressed IT recruitment challenges by developing a guide describing a 12-step process to improve visibility and popularity of recruitment messages. The goal of this guide was to increase the interest of potential participants and to offer researchers ways in which to anticipate and respond when IT communication difficulties arise. Recent work with Ramo and Prochaska [11] demonstrated the value of Facebook advertising as an effective mechanism to reach young adults in clinical research. However, reaching a target group under the age of 18 years imposes special issues. For example, although children can be attracted to recruitment advertising for research, they would still be required to obtain parental consent for participation. Although social networking may interest a child about a research project, additional means of informing and developing trust with a parent are still necessary. Sullivan et al [13] and Graham et al [12] each illustrated how changing the composition of banner advertising may improve communication to desired target groups. In the case of pediatric research, such customization may promote discourse between parents and children. The recruitment process, as described by Patel et al [21], is explained as a dialog or discourse that takes place between the investigator and the potential research participant. In the case of minor children, discourse needs to be promoted between investigator, parent, and child if pediatric recruitment is to be successful.

Our recent experience in recruiting boys for the Klinefelter syndrome study can be described as a multilayered strategy of communication using IT. The process of communication began with a traditional print exposure that proved to be ineffective. Adding the various IT communication approaches, including the study website, a computer-based research recruitment website, social networking on Facebook, exposure via support groups online, and by teleconference, offered parents multiple exposures to study information. Although our original sample size calculation called for 46 participants based upon an effect size of 0.40, the effect size from our study proved to be larger (–0.47). We believe that the overall number of participants (43 boys) did not negatively affect the study.

### Conclusions

Recruiting boys for a study on Klinefelter syndrome proved to be a challenging endeavor that was best accomplished using IT-based techniques. Important lessons were learned as we dealt with early recruitment challenges. The first lesson is that multiple exposures to the study information and personal contact with the researcher may be helpful in fostering parental trust. Parents must believe that the study, the institution, and the researcher are trustworthy before they will agree to have their child take part. These acts of communication, presented in multiple ways, were central to the success of our recruitment effort and are distinct advantages offered by IT-based strategies. Expenses related to website creation, registration of a domain name, website maintenance, and planning for social networking advertising were not initially anticipated by us, but should be considered by future researchers in the planning process when study budgets are developed. A limitation of our reported recruitment observations is that an in-depth recruitment analysis was not conducted to determine how multiple recruitment exposures occurred. Future IT-based recruitment efforts should preplan the collection of profile data, including IP addresses and tracking of how, when, and how many times a recruitment website was visited. This type of data may assist in the planning of customized approaches for the creation of more effective social networking banner advertising. Nevertheless, the observations from this study may advance the understanding of how difficult-to-recruit participants, like children, might be reached and have parental communication needs met with a view to obtaining their consent to participate in a study. It is noteworthy to mention that there has been inadequate representation of children in Klinefelter syndrome research and in other genetic conditions.

Researchers need to expand their knowledge of how potential recruits might be encouraged to participate in studies by understanding the utility of traditional approaches versus IT and other social networking approaches for recruitment. By offering multiple opportunities for exposure, parents have the opportunity to digest and think about the idea of having their child participate in a study. Because IT and social networking have become well-accepted modes of communication, these tools enable the researcher to layer the recruitment message in order to optimize the likelihood that recruitment efforts will be successful.

## Multimedia Appendix 1

Screenshot of Columbia University Klinefelter syndrome study website.

## Multimedia Appendix 2

Screenshot of Knowledge Support & Action (KS&A) Klinefelter syndrome advocacy association website.

## Multimedia Appendix 3

Screenshot of Klinefelter Syndrome Global Support Group website.

## Multimedia Appendix 4

Screenshot of RecruitSource website.

## Multimedia Appendix 5

Screenshot of PrivateAccess website.

## Multimedia Appendix 6

Screenshot of Klinefelter syndrome study Facebook advertisement.

## Abbreviations

IP: Internet protocol
IT: information technology
KS: Klinefelter syndrome
