# Supplementary figures and images for: Using Information Technology and Social Networking for Recruitment of Research Participants: Experience From an Exploratory Study of Pediatric Klinefelter Syndrome
Source: J Med Internet Res. 2013 Mar 19;15(3):e48. doi: 10.2196/jmir.2286 (PMC3636115; doi:10.2196/jmir.2286)

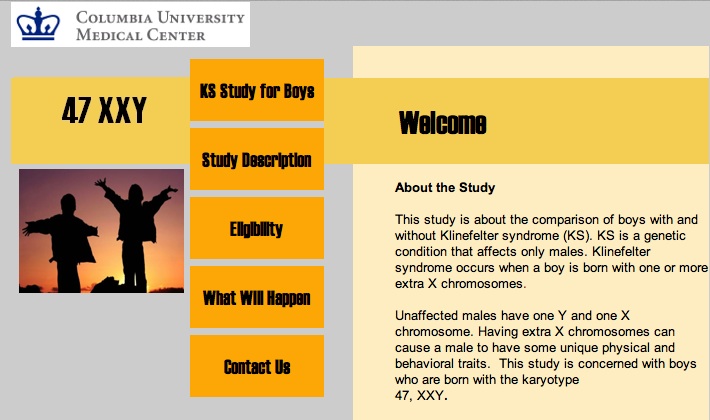

Supplement: Supplementary file 1 [file jmir_v15i3e48_app1.jpg]

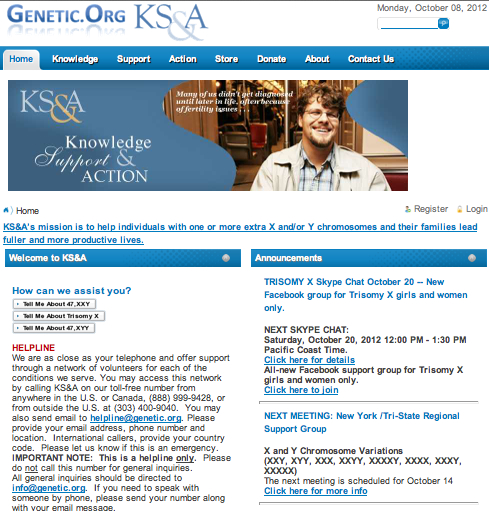

Supplement: Supplementary file 2 [file jmir_v15i3e48_app2.jpg]

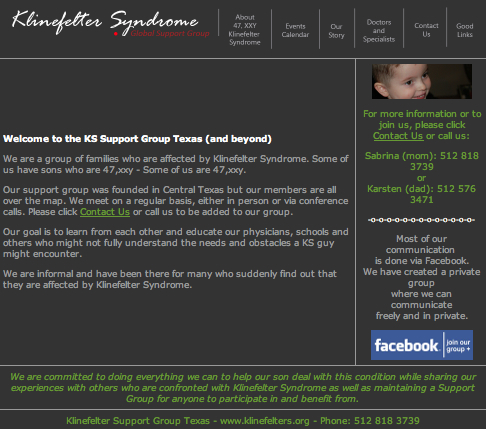

Supplement: Supplementary file 3 [file jmir_v15i3e48_app3.jpg]

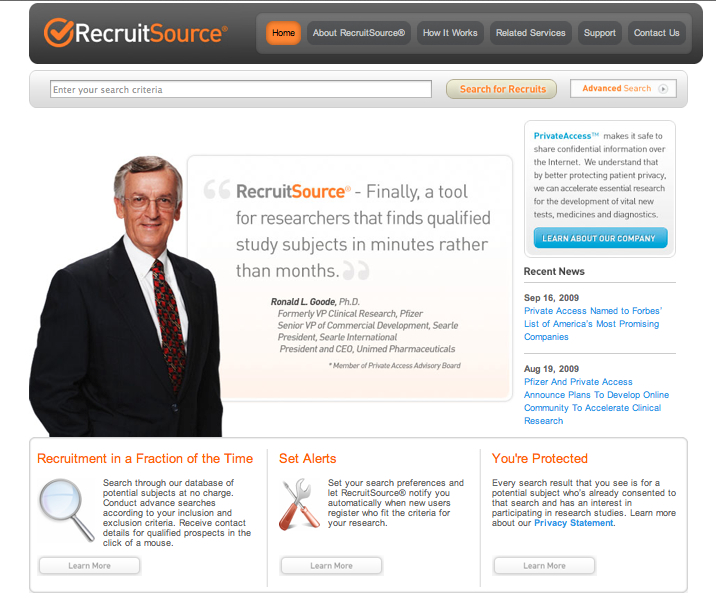

Supplement: Supplementary file 4 [file jmir_v15i3e48_app4.jpg]

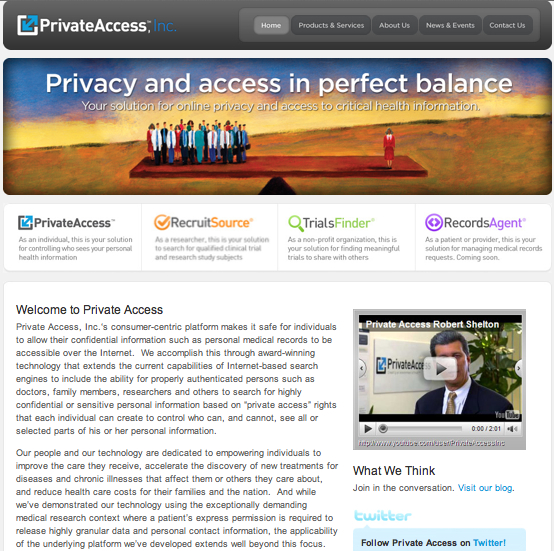

Supplement: Supplementary file 5 [file jmir_v15i3e48_app5.jpg]

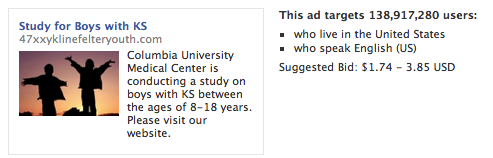

Supplement: Supplementary file 6 [file jmir_v15i3e48_app6.jpg]
